# Chronotype mediates gender differences in risk propensity and risk-taking

**DOI:** 10.1371/journal.pone.0216619

**Published:** 2019-05-23

**Authors:** Rebecca Gowen, Allan Filipowicz, Krista K. Ingram

**Affiliations:** 1 Biology Department, Colgate University, Hamilton, New York, United States of America; 2 S.C. Johnson College of Business, Cornell University, Ithaca, New York, United States of America; Universitá Cattolica del Sacro Cuore, ITALY

## Abstract

Risk-taking is a complex form of decision-making that involves calculated assessments of potential costs and rewards that may be immediate or delayed. Thus, making predictions about inter-individual variation in risk-taking due to personality traits, decision styles or other attributes can be difficult. The association of risk-taking with gender is well-supported; males report higher propensity for risk-taking and show higher risk-taking on tasks measuring actual risk-taking behavior. Risk-taking also appears to be associated with circadian phenotypes (chronotypes), with evening-types reporting higher levels of risk-taking—but this association may be confounded by the fact that, in certain age groups, males are more likely to be evening-types. Here, we test for gender by chronotype effects on risk-taking in young adults (n = 610) using a self-reported risk propensity questionnaire, the health domain of the DOSPERT, and a behavioral task measuring risk-taking, the Balloon Analog Risk Task (BART). Our results show that males report and take significantly more risks than females in this population. In addition, evening-type individuals have significantly higher self-reported risk propensity and tend to take more risks on the BART. Interestingly, there is no significant difference in risk propensity or risk-taking behavior across male circadian phenotypes, but evening-type females significantly report and take more risk than female intermediate and morning types. In regression analyses, we found both gender and chronotype predict risk propensity and risk-taking. Path analysis confirms that chronotype has an indirect effect on gender differences in both risk propensity and risk-taking. Furthermore, we found that trait anxiety (STAI) and sleep disturbance (PROMIS), significantly correlate with chronotype and gender in the complete dataset, but do not independently predict differences in female risk-taking. These results suggest that chronotype mediates gender effects on risk-taking and that these effects are driven primarily by morning-type females, but are not related to gender-specific differences in trait anxiety or sleep quality.

## Introduction

Individuals vary in the timing of internal circadian rhythms, resulting in variation in sleep-wake cycles (sleep-wake chronotypes) and/or preferences for timing of activities (diurnal preference) [1–5]. These circadian behavioral phenotype variants can be characterized into three general categories or chronotypes: morning types (MT), evening types (ET) and intermediate types (IT)[3]. Morning types tend to wake up early and prefer activities earlier in the day; evening types generally wake up later and prefer to time peak activity during the late afternoon or evening. An individual’s chronotype can vary with age, with morning chronotypes predominant in childhood and late adulthood, and a general transition to evening chronotypes during adolescence [6]. Females also tend to have earlier chronotypes compared to men until mid-life [6]. Inter-individual differences in circadian rhythms can influence human cognitive behavior and decision-making, but these effects vary widely across behaviors (reviewed in Adan et al. (2010)) [4].

Circadian phenotypes are associated with shifts in both molecular and physiological timing. The timing of individual circadian timing likely interacts with physiological pathways regulating energetic resources that influence cognitive processes, including self-control mechanisms [7–15]. These psycho-physiological effects on self-control are thought to be the mechanism by which chronotype affects decision-making, with individuals achieving synchrony or peak cognitive performance at their circadian peak. Support for synchrony effects has been found for some types of decision-making, like ethical choice tasks [16,17] customer service performance [18] and categorization [15,18,19] but not others, including creative problem solving [20]. Lack of energetic resources for self-regulation is also the basis for hypotheses involving social jetlag effects on cognitive processes; evening-type individuals are perpetually misaligned to the social and external environment and thus experience chronic pressures on self-regulation processes [13,21]. Proposed effects of social jetlag on ET include poor school performance and higher risk of mood disorders [22–24].

Risk-taking differs from other types of cognitive decision-making in that it involves an evaluation of potential rewards and costs, which can vary with context. Thus, risk-taking has been viewed as both a stable trait (propensity to take risks) and a state (actual risk-taking behavior) [25]. Relative to other types of decision-making, the effects of chronotype on risk may modulated by self-control mechanisms via the influence of personality traits, (rather than solely due to energetic resources). A growing body of literature suggests that eveningness is associated with the personality traits of impulsiveness and sensation seeking [7,25,26] and that risk-takers score higher in impulsiveness [27], though eveningness can influence risk-taking independent of personality [25]. In addition, risk-taking is associated with particular decision-making styles, including avoidant strategies and spontaneous decision-making (related to impulsivity personality) [26].

Limited previous research on circadian effects on risky decision-making suggests that chronotype affects both the propensity to take risks and risk-taking behavior in humans. Killgore (2007) split a sample of 54 young adults (29 male/25 female) into evening-types and morning types based on self-reported sleep patterns and found that evening types have a greater propensity for risk-taking on the Evaluation of Risks Scale, but did not take more risks on the Balloon Analog Risk Task (BART) [28]. It is not clear how gender influenced these results. Using a domain-specific measure of risk propensity, the Domain-Specific Risk-Taking (DOSPERT) Scale [29], and scenario-based tasks to evaluate risk-taking, Wang & Chartrand (2015) found a similar result; evening types scored higher on the financial portion of the DOSPERT only, but did not show significantly more risk-prone decisions on the scenario task [30]. The authors controlled for age and gender in their analysis, but did not report on gender-specific patterns in risk-taking with chronotype. A previous smaller study on college students in our lab showed no effect of self-reported chronotype on risk-taking behavior using the BART, but we did not consider gender differences in that analysis [17].

Although there are still gaps in our understanding of how gender moderates behavior, studies have shown that personality traits, like impulsivity, and their influence on behavior, can differ between genders [31–33]. Thus, gender may be a major confounding factor in how chronotype modulates risk-taking behaviors. Harris and colleagues (2006) found that females were less likely to make risky decisions, but only related to questions in domains involving gambling, recreation, and health [34]. In addition, males tend to report more risk-taking and take more risks than females [35]. Because a higher proportion of males in a young adult population are evening type, it is possible that higher risk propensity in ETs is explained, in part, by associative gender effects on risk-taking; i.e. males take more risks, more males are ET, thus ET take more risks. Gender effects may also interact with chronotype effects on risk-taking. In a recent study, Maestripieri (2014) measured differences between genders in relationship orientation and risk-taking propensity and found that eveningness was associated with willingness to take risks in females, but not in males, with risk-propensity of evening-type females resembling levels found in males [36].

In the current study, we test for gender and chronotype effects on risk-taking in a sample of young adults (n = 610), including undergraduate and graduate school students. We use an established risk propensity measure that is relevant to this particular population (the health domain of the DOSPERT), and a well-supported risk-taking task, the Balloon Analog Risk Task (BART). We characterize the circadian phenotypes of our participants with the full, self-reported, Horne-Ostberg Morningness-Eveningness questionnaire (HO-MEQ) and include intermediate chronotypes in our analysis. We test whether risk-propensity and risk-taking are associated with both chronotype and gender, and explore whether the relationship between chronotype and risk differs between genders.

## Materials and methods

### Participants

Undergraduate students at Colgate University and graduate students at Cornell SC Johnson College of Business volunteered to participate in the study (n = 610; 390 females, 220 males, average age 21.1 (±4.98) yrs; range 17–58). All methods adhered to the principles of the Declaration of Helsinki; the Institutional Review Board at Colgate University and Cornell University approved all procedures and consent forms (#FR-F13-07, #ER-F14-12, #ER-F15-13, #ER-F16-19, #ER-F17-13, #1504005518). All participants gave written informed consent. The requirement of parental consent for involvement of university students under the age of 18 in this study was waived by the Institutional Review Board at Colgate University.

Each participant took a computer-based survey with questions from the full (19-question) Horne-Östberg Morningness-Eveningness Questionnaire (MEQ), the trait version of the Spielberger’s State-Trait Anxiety Scale (STAI), the short form of the Patient-Reported Outcomes Measurement Information System (PROMIS) Sleep Disturbance and the health questionnaire portion of the DOSPERT, and then completed a Balloon Analog Risk Task (BART) [3,29,37–39].

### Circadian phenotyping

Horne-Östberg MEQ scores range from 30 (extreme morning preference) to 70 (extreme evening preference). Individuals were classified as follows: MEQ scores ≤41 were designated evening-types (ET), MEQ ≥ 59 were designated morning types (MT), and between 42–58 were intermediate types (IT).

### Risk measures

The Domain-Specific Risk Attitude Scale (DOSPERT) was used to measure participants’ propensity of risk-taking behavior [29]. This survey is a self-reported measure of intended risk in the domains of ethical, recreational, social, health and financial decisions. In this study, we used the health domain. Participants report how likely they are to engage in risky behavior relating to each of the questions regarding decisions in the health and safety domain.

Actual risk-taking behavior was measured using the Balloon Analog Risk Task (BART), a computerized measure of risk-taking behavior [37]. Participants pumped a virtual balloon and earned money in their account for each pump as the balloon inflated. If the balloon popped before the participants ‘cashed out’, they lost the money they earned in that round. The participants were given an incentive to bank money; six participants were randomly selected throughout the study to win the total of their earnings from the BART.

### Sleep disturbance and anxiety

Sleepiness is known to influence metabolic physiology and may modulate decision-making independent of circadian effects. Participants completed the short form PROMIS sleep disturbance questionnaire, allowing us to compare measures of risky decision-making with a standardized measure of sleep disturbance [39]. Scores generated from this survey represent a standardized score with a mean of 50 and SD of 10. Higher scores indicate poor sleep with values greater than 55 and 60 representing mild and severe sleep disturbance, respectively.

We measured Spielberger Trait Anxiety Scale (T-Anxiety from the STAI) which evaluates aspects of “anxiety proneness,” including general states of nervousness, worry, and tension [38].

### Statistical analysis

Differences between diurnal preference chronotypes (ET, MT, IT) and genders in risk propensity (DOSPERT scores) and behavioral risk-taking (BART scores) were tested with GLM ANOVAs. Post-hoc analyses were tested with Tukey’s post-hoc tests.

To investigate the relationships among the study variables, bivariate correlations were computed for Pearson’s coefficients. A hierarchical linear regression analysis was performed to test how gender, chronotype, trait anxiety, and sleep disturbance predicted risk propensity and/or risk-taking. Model 1 tested how gender predicts risk-taking, Model 2 tested the effects of gender and chronotype, Model 3 tested the effects of gender, chronotype and trait anxiety, and Model 4 included the final study variable, sleep disturbance. We performed two mediation analyses using the PROCESS macro [40,41] that tested the role of chronotype as mediator of the relationships between gender and risk propensity and/or risk-taking. A bootstrapping procedure (with 5000 bootstrap samples) was used; a 95% CI that does not include zero provides evidence of a significant indirect effect [42]. For estimates of effect sizes for indirect effect, Preacher & Kelley (2011) suggested the use of standardized indirect effect [43]. We used this convention to estimate the indirect effect of chronotype. All statistical analyses were performed in SPSS.

## Results

### Chronotype effects on risk-taking

**Fig 1:**
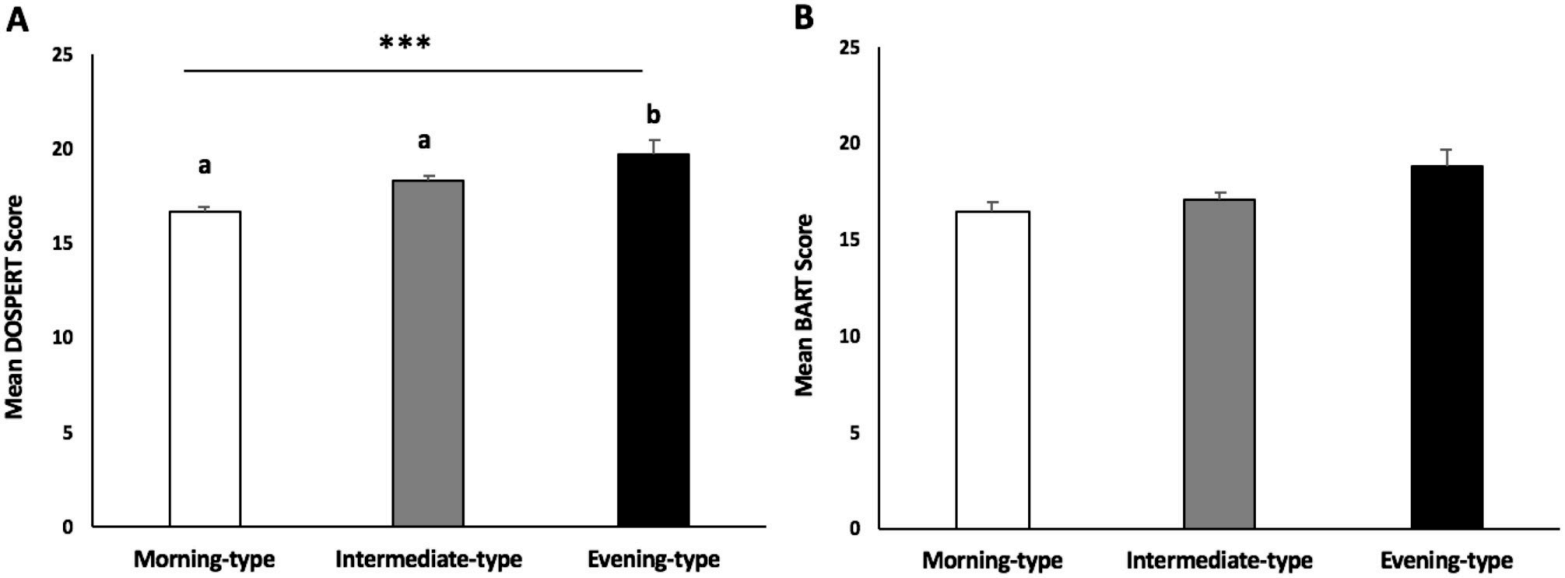
Differences in risk propensity and risk-taking behavior across diurnal preference chronotypes. **(A)** Risk propensity as measured by the score in the health/safety domain on the DOSPERT. Risk propensity was significantly different across diurnal preference groups (F(2,572) = 11.355, p<0.001). Tukey’s homogenous subsets show that evening types had greater perceived risk than morning or intermediate types. **(B)** Risk-taking behavior as measured by the mean number of balloon pumps on the BART. There were no significant effects of self-reported diurnal preference on BART scores (F(2,572) = 2.196, p = 0.112).

Risk propensity was significantly different across diurnal preference groups (F(2,572) = 11.355, p<0.001, Fig 1A). Tukey’s homogenous subsets show that evening types had greater perceived risk than morning or intermediate types. Although ETs tended to have higher risk-taking scores on the BART, this trend was not significant (F(2,572) = 2.196, p = 0.112; Fig 1B).

### Gender effects on risk-taking

**Fig 2:**
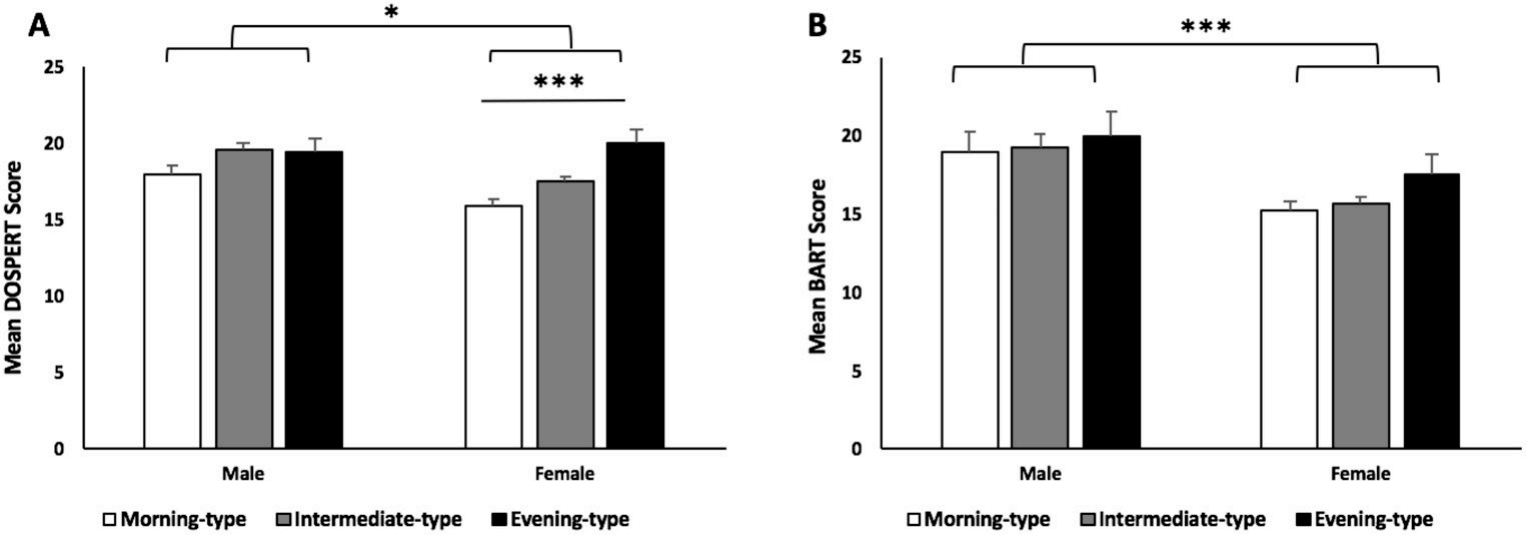
Risk-taking by gender and chronotype. Males and evening types report greater risk-taking; only females show significant differences in risk-taking across self-reported circadian phenotypes. **(A)** Males have greater risk propensity compared to females (F(1,570) = 5.160, p = 0.023) and evening-types overall have greater risk propensity than either intermediate or morning-types (F(2,570) = 9.097, p<0.001). Among females, evening types had significantly greater risk propensity than morning or intermediate types (F(2,358) = 11.331, p<0.001; Tukey’s homogenous subsets post-hoc analysis). **(B)** Risk-taking behavior on the BART was significantly higher in males than females (F(1,570) = 17.631, p<0.001). Female evening types show higher risk-taking if intermediates and morning types are pooled (t = 2.20, df = 356, p = 0.028).

Risk propensity was greater in males compared to females (F(1,570) = 5.160, p = 0.023; Table 1; Fig 2A). Within genders, risk propensity was significantly different across diurnal preference groups in females (F(2,358) = 11.331, p<0.001) but not males (F(2,212) = 1.908, p = 0.151). Tukey’s homogeneous subsets showed that female evening-types self-reported greater propensity for risky behavior than morning or intermediate females.

**Table 1:** Descriptive statistics for gender differences in risk propensity, risk-taking behavior, diurnal preference, anxiety, and sleep disturbance.

		Risk Propensity (DOSPERT)	Risk-taking (BART)	Chronotype (MEQ)	Trait Anxiety (STAI)	Sleep Disturbance (PROMIS)
**Male**	**Mean (SD)**	19.1 (5.05)	19.2 (8.73)	51.8 (10.3)	40.0 (9.51)	19.5 (5.58)
	**N**	219	214	220	220	217
**Female**	**Mean (SD)**	17.4 (4.79)	15.6 (6.28)	53.9 (9.76)	42.9 (10.9)	20.6 (5.93)
	**N**	390	360	389	386	376

Overall, males took significantly more risks on the BART than females (F(1,570) = 17.631, p<0.001; Fig 2B) but, within genders, differences between diurnal preference groups were not significant for either gender (F(2,570) = 1.078, p = 0.341).

### Correlates of risk-taking

Results of correlation analysis showed that both gender and chronotype were significantly and negatively correlated with risk propensity ((Pearson’s r_gender_ = -0.168, p<0.001); r_chronotype_ = -0.205, p<0.001; Table 2) and risk-taking ((Pearson’s r_gender_ = -0.232, p<0.001); r_chronotype_ = -0.108, p = 0.005; Table 3). Trait anxiety and sleep disturbance were not significantly related to risk-taking. However, gender and chronotype were significantly and negatively associated with trait anxiety ((Pearson’s r_gender_ = -0.131, p = 0.001); r_chronotype_ = -0.212, p<0.001) and sleep disturbance ((Pearson’s r_gender_ = -0.088, p = 0.017); r_chronotype_ = -0.467, p<0.001).

**Table 2:** Correlations of risk propensity, diurnal preference, anxiety, and sleep disturbance.

(n = 590)	Risk Propensity	Gender	Chronotype	Trait Anxiety	Sleep Disturbance
**Risk Propensity**	1.000	-0.168***	-0.205***	0.038	0.037
**Gender**		1.000	0.113**	0.131***	0.088*
**Chronotype (MEQ)**			1.000	-0.212***	-0.287***
**Trait Anxiety (STAI)**				1.000	0.467***
**Sleep Disturbance**‡					1.000

Pearson’s coefficient (r).

*p = 0.05,

**p<0.01

***p<0.001.

^‡^Sleep disturbance measured by PROMIS.

**Table 3:** Correlations of risk-taking behavior, diurnal preference, anxiety, and sleep disturbance.

(n = 560)	Risk-taking Behavior	Gender	Chronotype	Trait Anxiety	Sleep Disturbance
**Risk-taking (BART)**	1.000	-0.232***	-0.108***	-0.006	-0.016
**Gender**		1.000	0.126**	0.152***	0.097*
**Chronotype (MEQ)**			1.000	-0.215***	-0.291***
**Trait Anxiety (STAI)**				1.000	0.464***
**Sleep Disturbance**‡					1.000

Pearson’s coefficient (r).

*p = 0.05,

**p<0.01

***p<0.001.

^‡^Sleep disturbance measured by PROMIS.

Model 1 predicted that males will report and take more risks than females; our analysis supported this prediction with 3% of the variance explained by gender for risk propensity (β = −0.17, p = .000, Cohen’s d = 0.35) and 5.4% of the variance explained by gender for risk-taking on the BART (β = −0.23, p = .000, Cohen’s d = .51; Table 4). According to Cohen’s (1988) guidelines, these effect sizes are small to moderate [44]. Model 2 predicted that both gender and chronotype would provide a stronger prediction of risk-taking, with males and evening types reporting and taking more risks; we found that both gender and chronotype were equally robust predictors of risk propensity, explaining, in combination, 6% of the variance (β = −0.19, p = .000) and were slightly greater predictors for risk-taking than gender alone, together explaining 6% of the variance (β = −0.08, p = .050). Finally, inspection of regression coefficients for Models 3 and 4 did not reveal statistically significant additional effects for trait anxiety and sleep disturbance, explaining, in total, 6.4% of the variance for risk propensity and 6.1% for risk-taking.

**Table 4:** The effects of gender, diurnal preference, trait anxiety and sleep disturbance on risk propensity and risk-taking.

		Risk Propensity (DOSPERT)				Risk-taking (BART)			
	Predictors	R^2^	Adj R^2^	ß	*B* (95%CI)	R^2^	Adj R^2^	ß	*B*(95%CI)
**Step 1**	Gender	0.03	0.03***	-0.17	-1.74 (-2.57,-0.92)	0.05	0.05***	-0.23	-3.59 (-4.85,-2.34)
**Step 2**	Diurnal Preference	0.06	0.06***	-0.19	-0.10 (-0.14,-0.06)	0.06	0.06*	-0.08	-0.06 (-0.12,-0.00)
**Step 3**	Trait Anxiety	0.06	0.06	0.02	0.01 (-0.03, 0.05)	0.06	0.06	0.01	0.01 (-0.05, 0.07)
**Step 4**	Sleep Disturbance	0.06	0.06	-0.02	-0.01 (-0.10, 0.07)	0.06	0.06	-0.03	-0.04 (-0.16, 0.08)

Coefficients represent variables entered at each step of the hierarchical multiple regression analysis. Men are coded as 1; women, as 2. Coefficients represent variables entered at each step of the hierarchical multiple regression analysis.

AdjR2 p-values represent significant change in model at

*p = 0.05,

***p<0.001.

**Fig 3:**
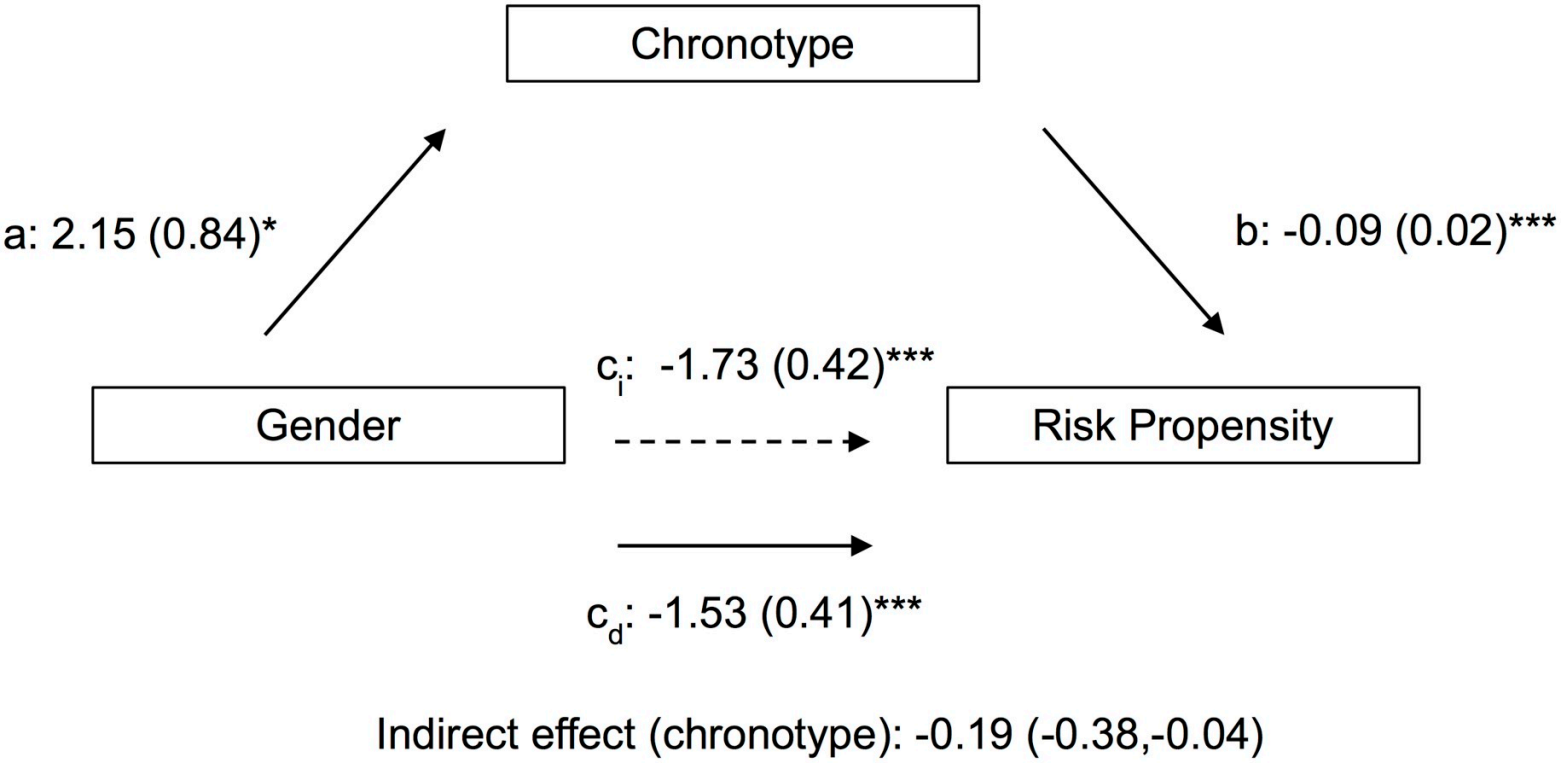
Path coefficients for mediation analysis of gender effects on risk propensity by chronotype. *a*, *b*, *c*_*i*_ and *c*_*d*_ are regression coefficients (males are coded as 1; females as 2; *i* subscript indicates indirect effect and *d* subscript indicates direct effect). *p<0.05, **p<0.01, ***p<0.001.

**Fig 4:**
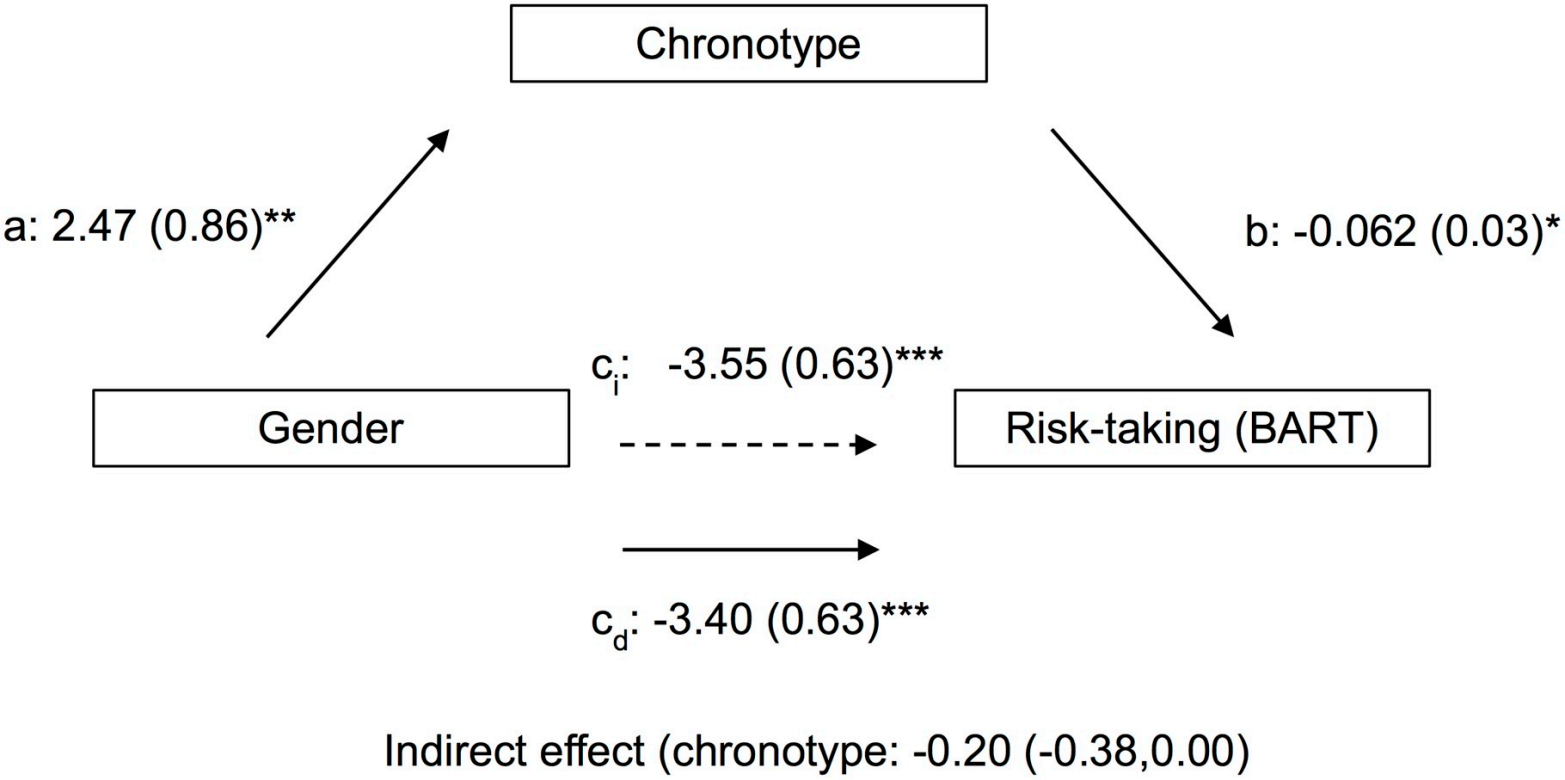
Path coefficients for mediation analysis of gender effects on risk-taking behavior by chronotype. *a*, *b*, *c*_*i*_ and *c*_*d*_ are regression coefficients (males are coded as 1; females as 2; *i* subscript indicates indirect effect and *d* subscript indicates direct effect). *p<0.05, **p<0.01, ***p<0.001.

The two mediation models estimated the total and direct effects of gender on risk propensity and risk-taking via mediation by indirect effects of chronotype (Figs 3 and 4). The risk propensity analysis revealed a significant negative indirect effect of gender on risk-taking through chronotype (point estimate = −0.19, 95% CI [−0.38, −0.04]). The BART risk-taking analysis also revealed a significant negative indirect effect of gender on risk-taking via chronotype (point estimate = −0.20, 95% CI [−0.38, −0.00]).

## Discussion

Our results provide robust support that evening-types report greater risk propensity in the health and safety domains, supporting the results of previous studies showing associations between eveningness and self-reported risk propensity in some DOSPERT domains [25,30]. Although previous studies did not find significant associations between chronotype and risk propensity in the health/safety domain, differences in populations sampled and/or the size of the study sample may explain the association of this domain in the current study. Individuals with self-reported evening preference in the current study also tended to take more risks in the BART behavioral task, but this association is weaker than for risk propensity. There is little support for associations between morningness-eveningness and risk-taking behavior on the BART in the literature [28], but scenario-based measures of financial risk-taking (gambling and/or investment) are negatively correlated with morningness [30].

In addition, our results reveal that gender is a significant factor in studies of chronotype effects on risk. Both chronotype and gender are significantly correlated with risk propensity and risk-taking behaviors. Adding the effects of chronotype to gender in the model significantly and robustly increases the prediction of risk propensity and moderately increases prediction of risk-taking behavior. Given the difference between genders in risk-taking across chronotype groups, we explored the mediation of gender effects on risk-taking by chronotype and measured the indirect effects of chronotype. Gender effects on both risk propensity and risk-taking behavior on the BART were mediated by chronotype, with indirect effects of moderate size.

Although males report greater risk-taking and take more risks than females in the current study, there is no association of chronotype with risk-taking in males. Instead, our results suggest that the correlation of eveningness and risk-taking is driven primary by differences between morning and evening-type females. In our study, evening-type females reported greater propensity for risk taking and displayed risk-taking behaviors similar to levels seen in males (see also [36]). The asymmetry in risk-taking propensity across genders has been well-documented [45], and the few studies that have tested for differences in risk-taking behavior among chronotypes have found conflicting results eg. [28,30]. Here, we show a chronotype effect on risk-taking that is limited to females, confirming the suggestion by Maestripieri (2014) that female evening-types display levels of risk similar to males [36]. Thus, it appears that morning (and intermediate) female chronotypes are more risk-averse than other chronotypes of both sexes. The outstanding question remains, why are females showing such differences in risk-taking across chronotype?

One of the leading hypotheses for high risk-taking in evening-types involves physiological processes related to circadian rhythms and social jetlag [28,30]. Social jetlag theory, proposed by Wittman et al (2006), explains how, in most social environments, evening-types must work against their internal clock [21]. One major effect of the misalignment of natural circadian rhythms with external demands is the mistiming of glucose metabolism [46] which, in turn, may impact energetic resources required for self-control [47]. Poor sleep quality may exacerbate circadian misalignment and also negatively affect self-control resources. Given that females in this population have higher sleep disturbance, we examined whether sleep disturbance might be a factor influencing gender differences in chronotype-specific risk-taking. However, we found that sleep quality was not a significant factor in predicting overall chronotype effects on risk-taking, nor was it a factor when only female participants were considered.

Gender differences in anxiety may also influence risk-taking; females tend to have higher levels of anxiety, and this population shows significantly higher anxiety levels in females relative to males. State anxiety has been shown to modulate gender differences in risk-taking [48] and trait anxiety has been linked to decreased risk-taking [49–53]. In this study, trait anxiety was correlated with chronotype and gender, but did not influence risk-taking independent of these factors. Thus, it is not clear why females, particularly morning-type females, appear to be so risk-averse, although state anxiety may play a role [48].

The strengths of this study include a large sample size and a test design that included both self-reported risk propensity and an actual risk-taking measure. We also used the full version of the Horne-Ostberg Morningness-Eveningness Questionnaire, which is a well-established criterion for measuring one aspect of circadian phenotype, diurnal preference. However, we did not include a measure of the sleep-wake chronotype as estimated from the Munich Chronotype Questionnaire. The study was limited in its age range as we sampled primarily young adults. Although our results apply to risk-taking in only a subset of the population, this subset includes individuals for which risk-taking is a serious public health concern. Our study includes only the health domain and we did not include other sub-domains of the DOSPERT. This may limit the interpretation of our results on risk propensity as they cannot be generalized for all domains of risk behavior. We did not include state anxiety or multiple risk propensity and risk-taking tasks.

## Conclusions

Our study provides strong support for the association between self-reported eveningness and higher risk propensity, as well as weaker support for higher risk-taking. We highlight a robust gender by chronotype effect where only females show a significant correlation between diurnal preference and risk-taking, with morning-type and intermediate-type females being more risk-averse than other individuals. Recent advances in chronobiology have highlighted major differences in how females and males respond physiologically to circadian factors [54,55]. These gender differences may extend to circadian effects on decision-making and on risk-taking, in particular.

## Supporting information

S1 Table Supporting data file. Complete data for this paper is available in Excel spreadsheet format.
(CSV)

## References

[1] Mongrain V, Lavoie S, Selmaoui B, Paquet J, Dumont M. Phase relationships between sleep-wake cycle and underlying circadian rhythms in morningness-eveningness. J Biol Rhythms. 2004;19(3):248–57. 10.1177/0748730404264365 15155011

[2] Adan A, Natale V. Gender differences in morningness-eveningness preference. Chronobiol Int. 2002;19(4):709–20. 12182498 10.1081/cbi-120005390

[3] Horne JA, Ostberg O. A self-assessment questionnaire to determine morningness-eveningness in human circadian rhythms. Int J Chronobiol. 1976;4(2):97–110. 1027738

[4] Adan A, Archer SN, Hidalgo MP, Di Milia L, Natale V, Randler C. Circadian typology: A comprehensive review. Vol. 29, Chronobiology International. 2012 p. 1153–75. 10.3109/07420528.2012.719971 23004349

[5] Roenneberg T, Wirz-Justice A, Merrow M. Life between clocks: Daily temporal patterns of human chronotypes. J Biol Rhythms. 2003;18(1):80–90. 10.1177/0748730402239679 12568247

[6] Roenneberg T, Kuehnle T, Juda M, Kantermann T, Allebrandt K, Gordijn M, et al Epidemiology of the human circadian clock. Sleep Med Rev. 2007;11(6):429–38. 10.1016/j.smrv.2007.07.005 17936039

[7] Adan A, Natale V, Caci H, Prat G. Relationship between circadian typology and functional and dysfunctional impulsivity. Chronobiol Int. 2010;27(3):606–19. 10.3109/07420521003663827 20524804

[8] Caci H, Robert P, Boyer P. Novelty seekers and impulsive subjects are low in morningness. Eur Psychiatry. 2004;19(2):79–84. 10.1016/j.eurpsy.2003.09.007 15051106

[9] Randler C. Proactive people are morning people. J Appl Soc Psychol. 2009;39(12):2787–97.

[10] Muraven M, Baumeister RF. Self-Regulation and Depletion of Limited Resources: Does Self-Control Resemble a Muscle? Psychol Bull. 2000;126(2):247–59. 10748642 10.1037/0033-2909.126.2.247

[11] Randler C, Saliger L. Relationship between morningness-eveningness and temperament and character dimensions in adolescents. Pers Individ Dif. 2011;50(2):148–52.

[12] Tonetti L, Adan A, Caci H, De Pascalis V, Fabbri M, Natale V. Morningness-eveningness preference and sensation seeking. Eur Psychiatry. 2010;25(2):111–5. 10.1016/j.eurpsy.2009.09.007 19926258

[13] Digdon NL, Howell AJ. College students who have an eveningness preference report lower self-control and greater procrastination. Chronobiol Int. 2008;25(6):1029–46. 10.1080/07420520802553671 19005903

[14] Stolarski M, Ledzińska M, Matthews G. Morning is tomorrow, evening is today: Relationships between chronotype and time perspective. Vol. 44, Biological Rhythm Research. 2013 p. 181–96.

[15] May CP. Synchrony effects in cognition: The costs and a benefit. Psychon Bull Rev. 1999;6(1):142–7. 12199309 10.3758/bf03210822

[16] Gunia BC, Barnes CM, Sah S. The Morality of Larks and Owls: Unethical Behavior Depends on Chronotype as Well as Time of Day. Vol. 25, Psychological Science. 2014 p. 2272–4. 10.1177/0956797614541989 25287664

[17] Ingram KK, Ay A, Bin Kwon S, Woods K, Escobar S, Gordon M, et al Molecular insights into chronotype and time-of-day effects on decision-making. Sci Rep. 2016;6. 10.1038/srep29392 PMC4937423 27388366

[18] Hornik J, Miniero G. Synchrony effects on customers’ responses and behaviors. Int J Res Mark. 2009;26(1):34–40.

[19] Yang L, Hasher L, Wilson DE. Synchrony effects in automatic and controlled retrieval. Psychon Bull Rev. 2007;14(1):51–6. 10.3758/bf03194027 17468781 PMC1858628

[20] Wieth MB, Zacks RT. Time of day effects on problem solving: When the non-optimal is optimal. Think Reason. 2011;17(4):387–401.

[21] Wittmann M, Dinich J, Merrow M, Roenneberg T. Social jetlag: Misalignment of biological and social time. In: Chronobiology International. 2006 p. 497–509. 10.1080/07420520500545979 16687322

[22] Giannotti F, Cortesi F, Sebastiani T, Ottaviano S. Circadian preference, sleep and daytime behaviour in adolescence. J Sleep Res. 2002;11(3):191–9. 12220314 10.1046/j.1365-2869.2002.00302.x

[23] Haraszti RÁ, Ella K, Gyöngyösi N, Roenneberg T, Káldi K. Social jetlag negatively correlates with academic performance in undergraduates. Chronobiol Int. 2014;31(5):603–12. 10.3109/07420528.2013.879164 24491157

[24] Levandovski R, Dantas G, Fernandes LC, Caumo W, Torres I, Roenneberg T, et al Depression scores associate with chronotype and social jetlag in a rural population. Chronobiol Int. 2011;28(9):771–8. 10.3109/07420528.2011.602445 21895489

[25] Ponzi D, Wilson MC, Maestripieri D. Eveningness is Associated with Higher Risk-Taking, Independent of Sex and Personality. Psychol Rep [Internet]. 2014;115(3):932–47. Available from: http://journals.sagepub.com/doi/10.2466/19.12.PR0.115c28z5 25457099 10.2466/19.12.PR0.115c28z5

[26] Tonetti L, Fabbri M, Boreggiani M, Guastella P, Martoni M, Ruiz Herrera N, et al Circadian preference and decision-making styles. Biol Rhythm Res. 2016;47(4):573–81.

[27] Butler GKL, Montgomery AMJ. Impulsivity, risk taking and recreational “ecstasy” (MDMA) use. Drug Alcohol Depend. 2004;76(1):55–62. 10.1016/j.drugalcdep.2004.04.003 15380289

[28] Killgore WDS. Effects of Sleep Deprivation and Morningness-Eveningness Traits on Risk-Taking “,.” Psychol Reports O Psychol Reports. 2007;100:613–26. 10.2466/pr0.100.2.613-626 17564238

[29] Blais A-R, Weber EU. A domain-specific risk-taking (DOSPERT) scale for adult populations. Judgm Decis Mak. 2006;1:33–47.

[30] Wang L, Chartrand TL. Morningness–Eveningness and Risk Taking. J Psychol [Internet]. 2015 5 19;149(4):394–411. Available from: 10.1080/00223980.2014.885874 25901637

[31] Weafer J, de Wit H. Sex differences in impulsive action and impulsive choice. Addict Behav. 2014;39(11):1573–9. 10.1016/j.addbeh.2013.10.033 24286704 PMC4012004

[32] Farstad SM, Von Ranson KM, Hodgins DC, El-Guebaly N, Casey DM, Schopflocher DP. The influence of impulsiveness on binge eating and problem gambling: A prospective study of gender differences in Canadian adults. Psychol Addict Behav. 2015;29(3):805–12. 10.1037/adb0000069 25961146

[33] Weinstein A, Dannon P. Is Impulsivity a Male Trait Rather than Female Trait? Exploring the Sex Difference in Impulsivity. Curr Behav Neurosci Reports [Internet]. 2015;2(1):9–14. Available from: http://link.springer.com/10.1007/s40473-015-0031-8

[34] Harris CR, Jenkins M, Glaser D. Gender Differences in Risk Assessment: Why do Women Take Fewer Risks than Men? Judgm Decis Mak. 2006;1(1):48–63.

[35] Reniers RLEP, Murphy L, Lin A, Bartolomé SP, Wood SJ. Risk perception and risk-taking behaviour during adolescence: The influence of personality and gender. PLoS One [Internet]. 2016;11(4):1–14. Available from: 10.1371/journal.pone.0153842 PMC4839773 27100081

[36] MaestripieriD. Night owl women are similar to men in their relationship orientation, risk-taking propensities, and cortisol levels: Implications for the adaptive significance and evolution of eveningness. Evol Psychol. 2014;12(1):130–47. 24566433

[37] Lejuez CW, Richards JB, Read JP, Kahler CW, Ramsey SE, Stuart GL, et al Evaluation of a behavioral measure of risk taking: The balloon analogue risk task (BART). J Exp Psychol Appl. 2002;8(2):75–84. 12075692 10.1037//1076-898x.8.2.75

[38] Spielberger CD. Manual for State-Trait Anxiety Inventory. Palo Alto, CA Consult Psychol Press 1983;

[39] Yu L, Buysse DJ, Germain A, Moul DE, Stover A, Dodds NE, et al Development of Short Forms From the PROMIS™ Sleep Disturbance and Sleep-Related Impairment Item Banks. Behav Sleep Med. 2011;10(1):6–24. 10.1080/15402002.2012.636266 22250775 PMC3261577

[40] Hayes AF. Beyond Baron and Kenny: Statistical Mediation Analysis in the New Millennium. Commun Monogr [Internet]. 2009;76(4):408–20. Available from: http://www.tandfonline.com/doi/abs/10.1080/03637750903310360

[41] Hayes AF. Introduction to Mediation, Moderation and Conditional Process Analysis. Vol. 53, Journal of Chemical Information and Modeling. 2013 527 p.

[42] Preacher KJ, Hayes AF. Asymptotic and resampling strategies for assessing and comparing indirect effects in multiple mediator models. In: Behavior Research Methods. 2008 p. 879–91. 18697684 10.3758/brm.40.3.879

[43] Preacher KJ, Kelley K. Effect size measures for mediation models: Quantitative strategies for communicating indirect effects. Psychol Methods. 2011;16(2):93–115. 10.1037/a0022658 21500915

[44] Cohen J. Statistical Power Analysis for the Behavioural Science (2nd Edition). In: Statistical Power Anaylsis for the Behavioural Science (2nd Edition). 1988 p. 25–7.

[45] Byrnes JP, Miller DC, Schafer WD. Gender differences in risk taking: A meta-analysis. Psychol Bull. 1999;125(3):367–83.

[46] Trenell MI, Marshall NS, Rogers NL. Sleep and metabolic control: Waking to a problem? Clin Exp Pharmacol Physiol. 2007;34(1–2):1–9. 10.1111/j.1440-1681.2007.04541.x 17201728

[47] Gailliot MT, Baumeister RF. The physiology of willpower: Linking blood glucose to self-control. Personal Soc Psychol Rev. 2007;11(4):303–27. 10.1177/1088868307303030 18453466

[48] Panno A, Donati MA, Milioni M, Chiesi F, Primi C. Why Women Take Fewer Risk Than Men Do: The Mediating Role of State Anxiety. Sex Roles. 2018;78(3–4):286–94.

[49] Giorgetta C, Grecucci A, Zuanon S, Perini L, Balestrieri M, Bonini N, et al Reduced risk-taking behavior as a trait feature of anxiety. Emotion. 2012;12(6):1373–83. 10.1037/a0029119 22775123

[50] Maner JK, Richey JA, Cromer K, Mallott M, Lejuez CW, Joiner TE, et al Dispositional anxiety and risk-avoidant decision-making. Pers Individ Dif [Internet]. 2007;42(4):665–75. Available from: 10.1016/j.paid.2006.08.016

[51] Maner JK, Schmidt NB. The Role of Risk Avoidance in Anxiety. Behav Ther. 2006;37(2):181–9. 10.1016/j.beth.2005.11.003 16942970

[52] Mitte K. Anxiety and risky decision-making: The role of subjective probability and subjective costs of negative events. Pers Individ Dif. 2007;43(2):243–53.

[53] Miu AC, Heilman RM, Houser D. Anxiety impairs decision-making: Psychophysiological evidence from an Iowa Gambling Task. Biol Psychol. 2008;77(3):353–8. 10.1016/j.biopsycho.2007.11.010 18191013

[54] Bailey M, Silver R. Sex differences in circadian timing systems: Implications for disease. Vol. 35, Frontiers in Neuroendocrinology. 2014 p. 111–39. 10.1016/j.yfrne.2013.11.003 PMC4041593 24287074

[55] Santhi N, Lazar AS, McCabe PJ, Lo JC, Groeger JA, Dijk D-J. Sex differences in the circadian regulation of sleep and waking cognition in humans. Proc Natl Acad Sci [Internet]. 2016;113(19):E2730–9. Available from: http://www.pnas.org/lookup/doi/10.1073/pnas.1521637113 27091961 10.1073/pnas.1521637113 PMC4868418

